# Challenges encountered during the management of pediatric facial soft tissue injury from a mobile battery blast: A case report

**DOI:** 10.1002/ccr3.4753

**Published:** 2021-08-30

**Authors:** Rikta Pande, Bandana Koirala, Mehul Jaisani, Chandrakant Pasvan

**Affiliations:** ^1^ Department of Pedodontics and Preventive Dentistry B.P. Koirala Institute of Health Sciences Dharan Nepal; ^2^ Department of Oral and Maxillofacial Surgery B.P. Koirala Institute of Health Sciences Dharan Nepal

**Keywords:** facial soft tissue injuries, lateral aesthetic unit of cheek, mobile battery blast, parotid gland, pediatric maxillofacial injuries

## Abstract

In case of parotid gland injury, one has to be aware of the fact that post‐operative complications like duct injury, leakage are very common. At times, a simple conservative approach turns out to be effective in managing complications of this nature.

## Graphical abstract

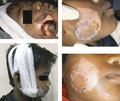

## 1 INTRODUCTION

Soft tissue injuries account for the majority of pediatric maxillofacial injuries. Although rarely life‐threatening, the treatment of these injuries can sometimes be complicated and may have a significant impact on the patient's facial function and aesthetics. There are multiple etiologies for trauma where socio‐demographic, cultural, and environmental factors play an important role in its occurrence. The present case reports facial soft tissue injury inflicted upon a 10‐year‐old boy from the disposed mobile battery blast on a roadside campfire. The injury involved the lateral aesthetic unit of the cheek, where the presence of the parotid gland and its duct can complicate the surgical management if a careful and early assessment of the wound and use of appropriate surgical techniques are not followed. The present case reports one of the challenges encountered from injury to the parotid gland and its management.

Facial trauma in children is a relatively common occurrence. In the present case, the facial injury resulted from the disposed mobile battery blast on a roadside campfire and involved the lateral aesthetic unit of the cheek, which represents the region between the buccal unit and auricle. This unit is covered with skin that adheres more to the underlying fascia hence can complicate the re‐construction.^1^ Concurrently, deep to the dermal layer there are delicate anatomical structures such as the parotid gland and facial nerves, which can complicate the surgical management if an early assessment of the wound and use of appropriate surgical techniques are not followed.

## 2 CASE REPORT

A 10‐year‐old male child was reported to the Pediatric emergency department of B.P. Koirala Institute of Health Sciences with a history of mobile battery blast injury on the right side of the face 6 h after the incident. There was a negative history of loss of consciousness and nausea/vomiting following the incident. Past medical and drug history were non‐significant.

### 2.1 On examination

**FIGURE 1:**
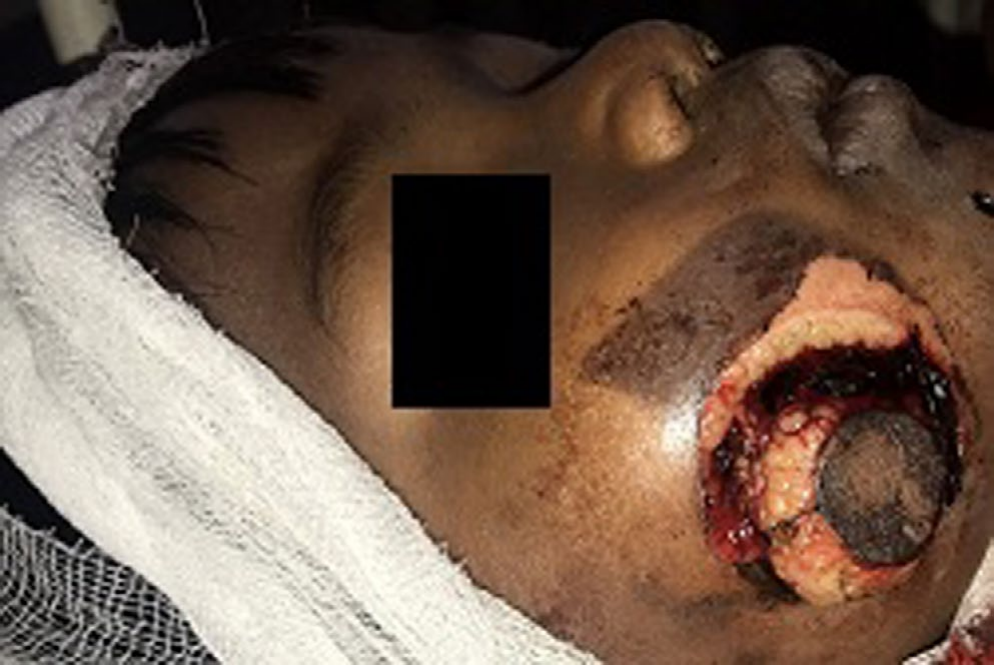
Initial presentation at Pediatric Emergency

Extraorally on the right lateral unit of the cheek, there was a circular injury of approximately 3 centimeters in diameter which was communicating intraorally to the oral cavity. There was herniation of the buccal pad of fat, blood clots, and superficial burn around the wound (Figure 1). Intraorally, on the right buccal mucosa laceration of around one centimeter along with the herniated buccal pad of fat was present. The parotid gland appeared to be injured; however, facial and trigeminal nerve functions were intact. Also, Stenson's duct was found to be patent when examined intraorally. Salivary flow could be detected from the Stenson's duct when manipulated intraorally as well as when the duct was cannulated from its distal oral opening with a pediatric intravenous catheter.

No injuries were present on other body parts. Based upon the findings, a clinical diagnosis of soft tissue injury^2^ was made.

### 2.2 Management

The patient was kept under antibiotic and analgesic therapy with injection Ceftriaxone (750 mg/BD) and injection Ketorolac (20 mg/TDS). The patient also received a booster dose of tetanus toxoid immunoglobulin.

A multidisciplinary team of Oral and Maxillofacial Surgery and the department of Pedodontics worked under general anesthesia for primary wound closure in an emergency operation theater. Initially, cleansing and rinsing of the wound were done using povidone‐iodine and normal saline solution, followed by marginal necrosectomy and debridement. Wound closure was done at multiple layers. Suturing of the oral mucosa, parotid gland, parotid capsule, and sub‐dermal layer was done with 4–0 polyglactin suture while 5–0 prolene suture was used on the superficial skin layer.

Post‐operatively, the patient was supported with antibiotics, analgesics, and fluid supplements, and the dressing was done twice a day for 1 week.

### 2.3 Post‐operative complications

**FIGURE 2:**
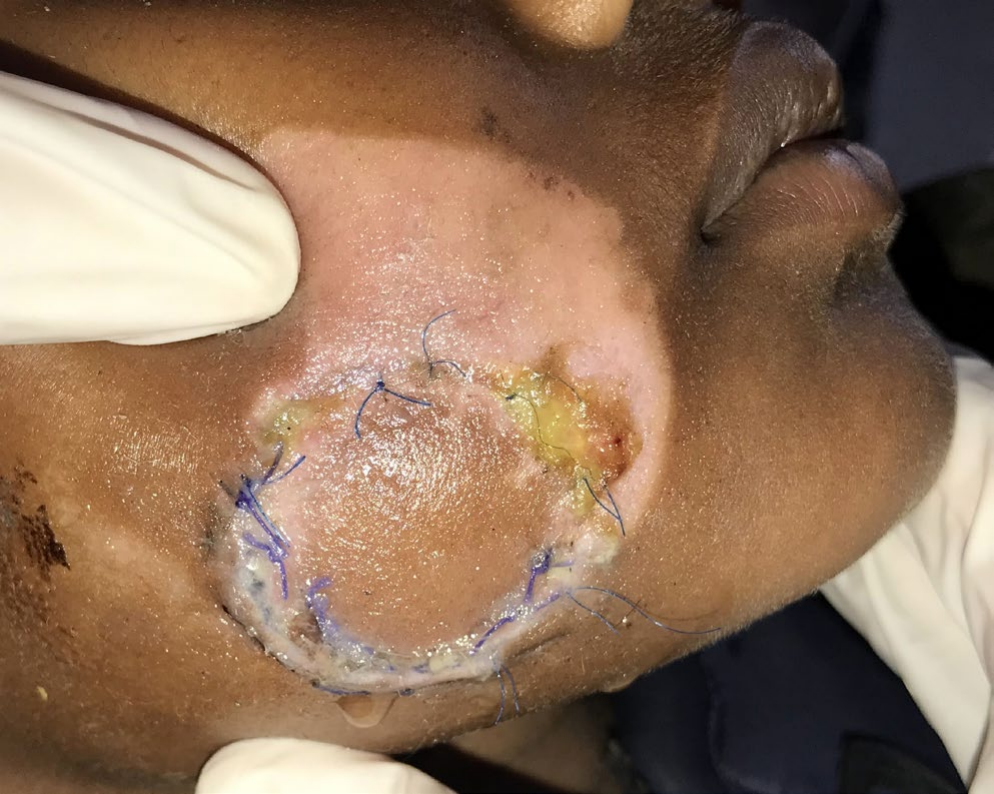
Post‐operative complication (Salivary leakage) on the fourth day

**FIGURE 3:**
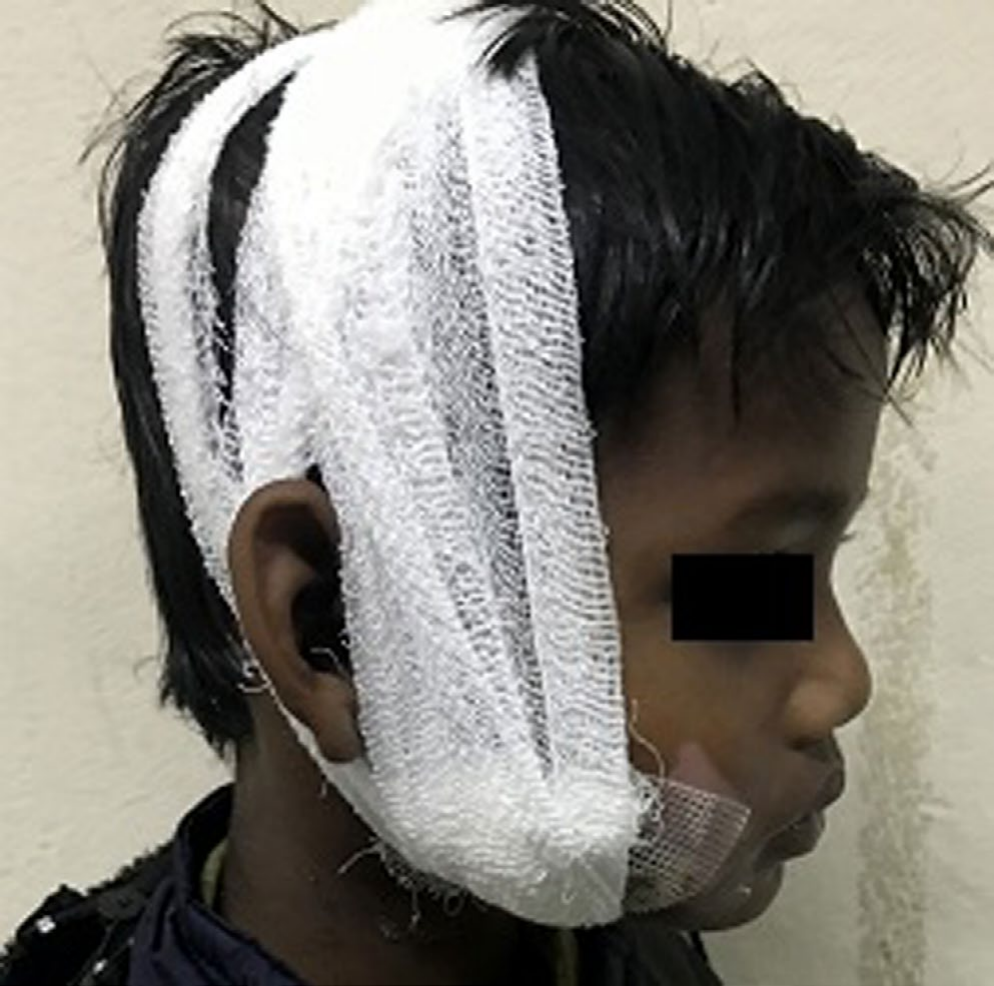
Pressure dressing

**FIGURE 4:**
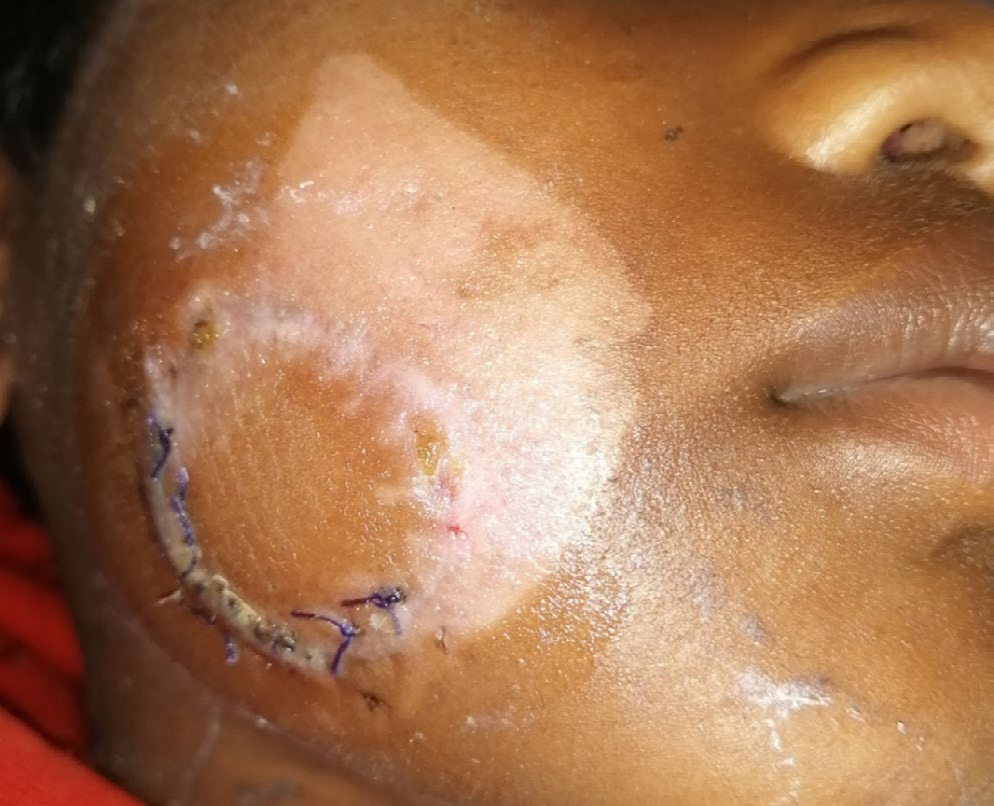
Fourteen post‐operative day (Stoppage of salivary leakage and satisfactory wound healing)

On the fourth post‐operative day, the patient developed salivary leakage from the extraoral margins of the sutured wound (Figure 2). In this case, the parenchyma of the gland was damaged along with rupture of the capsule while Stenson's duct was patent. An early attempt to repair the parenchyma of the capsule of the parotid gland was made at the time of initial surgery. However, salivary leakage was evident from the sutured site on fourth post‐operative day. Stenson's duct was re‐evaluated through cannulation but was found to be intact. Hence, conservative management via pressure packing was opted (Figure 3). Initially, the leakage was attempted to be managed using pressure dressing. Also, re‐suturing of the margins was performed under local anesthesia at the drainage site on the seventh post‐operative day. Pressure dressing was continued for 1 week which resulted in a stoppage of the leakage along with the recovery of the patient. Finally, on the fourteenth post‐operative day, sutures were removed and the patient was discharged in a healthy state (Figure 4).

## 3 DISCUSSION

Facial soft tissue injuries are a common occurrence in the pediatric population. Although rarely life‐threatening, treatment of these injuries can be complex and may have a significant impact on the patient's facial function and aesthetics.^1^

Management principles for soft tissue injuries in children are almost similar to those of adults. In this case, initial wound care was started with copious irrigation and debridement of the devitalized and necrotic tissues. Wound repair was done in multiple layers. Absorbable 4/0 or 5/0 vicryl or polydioxanone sutures (PDS) are suitable for muscle and subcutaneous tissue. Whereas, prolene/nylon 6/0 is the choice for skin approximation.^3^

Cheeks being the largest subunit of the face, this size correlates with a high frequency of injury to the cheeks and its underlying structures.^1^ Parotid duct injury should be considered in any deep injuries to the cheek area located inferior to a line extending from the tragus to the upper lip. Early detection and repair of ductal injuries are strongly advised since complications from delayed identifications are challenging. The signs of unrecognized parotid gland injury can be erythema, edema, sialocele, and tenderness after uneventful soft tissue repair.^4^

Post‐traumatic salivary fistulas, sialoceles, or leakages are initially managed with conservative treatment which includes pressure dressing, needle aspiration, and administration of broad‐spectrum antibiotics. A series by Lewis and Knottenbelt^5^ concluded that conservative management was adequate for parotid duct injuries. Similarly, Landau and Stewart^6^ concluded that systemic Probanthine, intravenous fluids, nil by mouth regimen, and external pressure would produce a resolution of symptoms, even in the presence of a fistula or sialocele. In the current case also, re‐suturing at the drainage site followed by pressure dressing for 1 week brought about the favorable result.

The present case also raised our attention toward the mishaps from the electronic wastes. Hence, preventive measures and education to the individual, parents, and society as a whole regarding the etiologies of trauma in children and the proper disposal/ re‐cycling of electronic wastes become very crucial.

## 4 CONCLUSIONS

Parotid gland injury should always be considered whenever a case of deep subcutaneous injury on the cheek region is encountered. Parotid gland was involved in the present case of facial trauma as well and the complication of salivary leakage arising from the ruptured parenchyma and parotid capsule in the case was detected and managed successfully using the conservative approach of compression dressing.

## CONFLICT OF INTEREST

We declare that there is no conflict of interest concerning to this article.

## AUTHOR CONTRIBUTIONS

Rikta Pande: Patient management and manuscript write‐up. Chandrakant Pasvan and Mehul R Jaisani: Primary patient management and patient follow‐up. Bandana Koirala: Manuscript editing and proofreading.

## ETHICAL APPROVAL

The patient has provided permission to publish the features of his case, and the identity of the patient has been protected. Informed consent has been obtained from the child and the parent.

## Data Availability

The data that supports the findings of this study are available on request from the corresponding author.
